# Anatomopathological features of uterine tube adenoma in a female dog

**DOI:** 10.29374/2527-2179.bjvm010626

**Published:** 2026-10-02

**Authors:** Lorrayne dos Santos Miranda, Marina Possa dos Reys, Lara Coslop Comerio, Rodrigo Viana Sepulveda, Lidianne Narducci Monteiro, Geovanni Dantas Cassali, Mayra Cunha Flecher

**Affiliations:** 1 Department of Veterinary Pathology, Universidade Vila Velha (UVV), Vila Velha, ES, Brazil.; 2 Department of General Pathology, Laboratory of Comparative Pathology, Universidade Federal de Minas Gerais (UFMG), Belo Horizonte, MG, Brazil.; 3 Department of Small Animal Surgery and Clinical Medicine, Universidade Vila Velha (UVV), Vila Velha, ES, Brazil.

**Keywords:** neoplasm, uterus, dog, reproductive system, neoplasia, útero, cão, sistema reprodutor

## Abstract

The uterine tube is part of the female reproductive system responsible for connecting the ovary to the uterus. Neoplasms originating from the uterine tube are rare in both animals and humans, with few reports available in the veterinary literature. A 7-year-old mixed-breed bitch was presented with clinical signs of lethargy, weight gain, and abdominal distension. Ultrasonography and computed tomography revealed a large abdominal mass. The patient underwent exploratory laparotomy for surgical excision of the mass, which was found adhered to the right ovary. Grossly, the mass measured 27.5 × 22.5 × 5.5 cm, had a soft consistency, irregular contours, reddish coloration, and a papillary appearance. Histopathological evaluation revealed well-differentiated papillary projections lined by ciliated columnar epithelial cells, with positive expression of estrogen and progesterone receptors and low Ki-67 labeling index, supporting the diagnosis of uterine tube adenoma. Although these tumors may reach considerable dimensions, they are generally associated with a favorable prognosis. In the present case, the diagnosis was established based on the correlation of clinical, morphological, and histopathological findings.

## Resumo

A tuba uterina é parte do sistema reprodutor feminino responsável por conectar o ovário ao útero. As neoplasias originadas da tuba uterina são raras tanto em animais quanto em humanos, havendo poucos relatos disponíveis na literatura veterinária. Uma cadela sem raça definida, de 7 anos de idade, foi atendida apresentando sinais de letargia, ganho de peso e distensão abdominal. A ultrassonografia e a tomografia computadorizada revelaram uma grande massa abdominal. A paciente foi submetida à laparotomia exploratória para excisão cirúrgica da massa, a qual se encontrava aderida ao ovário direito. Macroscopicamente, a massa media 27,5 × 22,5 × 5,5 cm, apresentava consistência macia, contornos irregulares, coloração avermelhada e aspecto papilífero. A avaliação histopatológica revelou projeções papilíferas bem diferenciadas, revestidas por células epiteliais cilíndricas ciliadas, com receptores de estrógeno e progesterona positivo e baixa expressão positiva para Ki-67 indicando o diagnóstico de adenoma de tuba uterina. Embora esses tumores possam atingir dimensões consideráveis, estão associados a prognóstico favorável. No presente caso, o diagnóstico foi estabelecido com base na correlação entre achados clínicos, morfológicos e histopatológicos.

## Introduction

Uterine tube tumors are uncommon in domestic mammals, and there are limited reports describing this neoplasm in the veterinary literature (Agnew & MacLachlan, 2016). Benign uterine tube tumors reported in animals have been referred to by various terms, including adenomas, papillary adenomas, fibroadenomas, adenomyomas, and adenomatoid tumors (Gelberg & McEntee, 1986; Marino et al., 2007; Minoccheri & Meluzzi, 1979; Pierezan et al., 2016; Sailasuta et al., 1989). However, it was proposed in veterinary pathology to unify the nomenclature for all benign epithelial tumors of the uterine tube under the term adenoma (Kennedy et al., 1998).

In avian species, particularly in chickens, tumors of the oviducts, which anatomically correspond to the uterine tube in mammals, are more frequently observed and have been more thoroughly studied (Anjum et al., 1989). In women, uterine tube neoplasms are rare (Hosokawa et al., 2006; Shahi et al., 2017), and sporadically reported in other species such as rabbits (Chambers et al., 2014).

Epidemiological surveys on female genital tract neoplasms in humans indicate that malignant tumors of the Fallopian tube account for only 0.1% to 0.5% of all female genital tumors, often associated with chronic inflammatory processes (Rose et al., 1990; Rosenblatt et al., 1989).

Given the rarity of uterine tube neoplasms in mammals, the present report aims to describe a case of uterine tube adenoma in a female dog, with emphasis on the gross and histopathological features of the lesion.

## Case description

A 7-year-old mixed-breed female dog was evaluated at the Veterinary Hospital of Vila Velha University, Espírito Santo, Brazil. According to the guardian, the dog exhibited signs of lethargy, weight gain, and progressive abdominal enlargement. Abdominal ultrasonography revealed a heterogeneous mass occupying a large portion of the abdominal cavity, with indeterminate origin and difficult measurement. Computed tomography subsequently confirmed the presence of a mass measuring approximately 23,9cm x 12,5cm x 23,1cm, causing mass effect on adjacent abdominal structures and exhibiting close contact with intestinal segments (Figure 1A).

**Figure 1:**
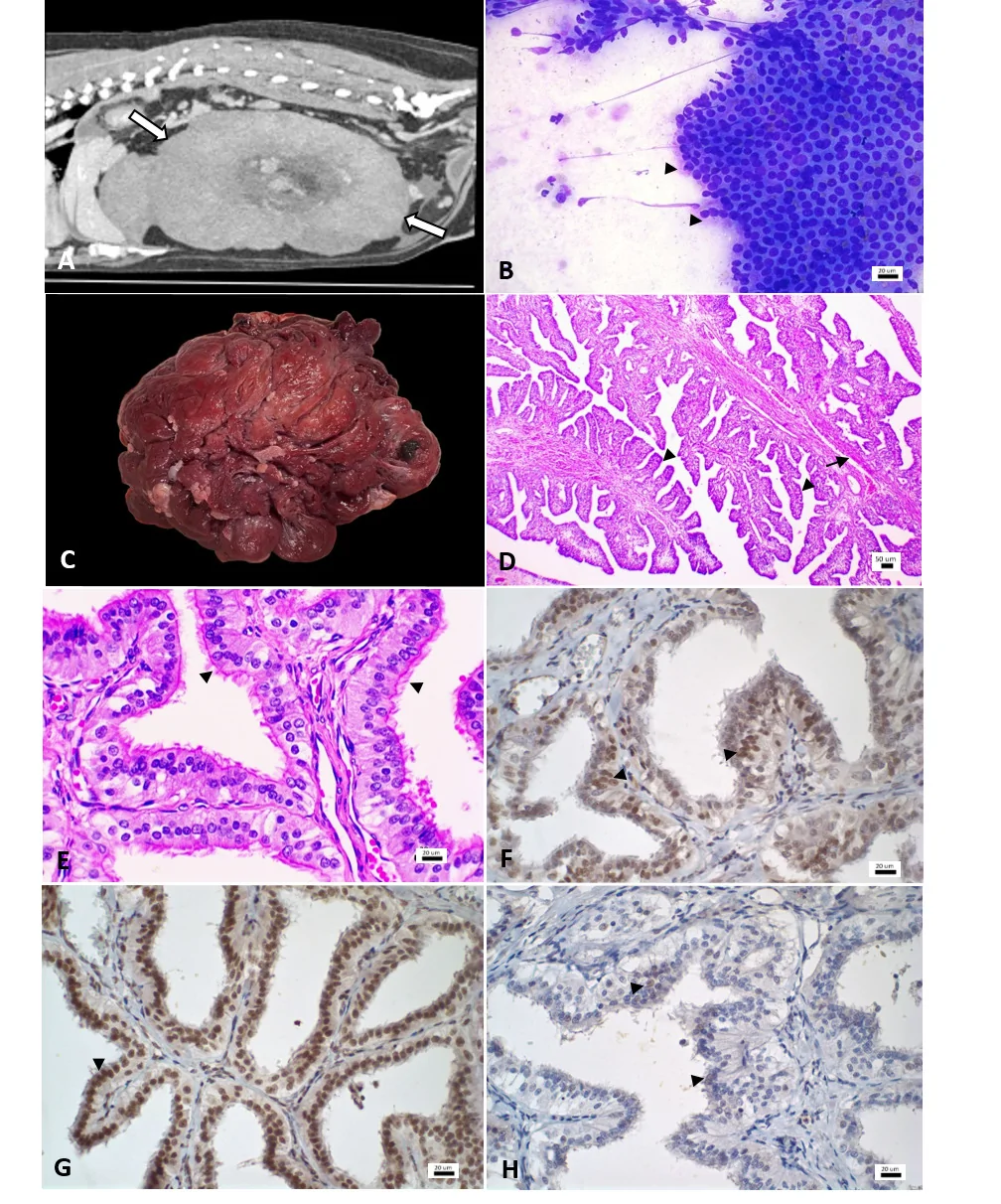
Female dog, uterine tube adenoma. **A.** Computed tomography. Lateral-lateral axis. Neoplasm with soft tissue density exerting mass effect on adjacent abdominal structures (arrows). **B.** Cytology of uterine tube adenoma. Smear composed of cohesive groups of ciliated epithelial cells (arrows), with round, homogeneous nuclei. Romanowsky stain, 400×(Bar 20um). **C.** Gross appearance. Irregular, reddish, papillary mass measuring 27.5 × 22.5 × 5.5 cm. **D.** Papillary projections supported by fibrovascular stroma, with occasional areas containing smooth muscle bundles (arrow). Hematoxylin and eosin stain, 40× Bar 50um). **E.** Papillary projections lined by one to two layers of well-differentiated ciliated columnar epithelial cells (arrowhead). Hematoxylin and eosin stain, 400×(Bar 20um). **F**. Immunohistochemistry for estrogen receptor (ER) demonstrated nuclear labeling in 80% of the cells (arrowhead). 400×(Bar 20um). **G**. Immunohistochemistry for estrogen receptor (PR) demonstrated nuclear labeling in 90% of the cells (arrowhead). 400×(Bar 20um). **H.** Immunohistochemistry for Ki-67 demonstrated nuclear labeling in fewer than 10% of the cells (arrowhead). 400×(Bar 20um).

Ultrasound-guided fine-needle aspiration cytology of the mass, in Romanowsky-type staining, revealed cohesive cellular islands composed of basophilic epithelial cells with scant, a homogeneous and occasionally vacuolated cytoplasm, and indistinct cell borders. Many cells exhibited structures resembling cilia. Nuclei were oval, basophilic, and occasionally contained visible nucleoli (Figure 1B). Mild anisocytosis, anisokaryosis, and cellular and nuclear pleomorphism were observed.

Following diagnostic imaging and cytology, the animal underwent exploratory laparotomy for excision of the mass. Intraoperative findings suggested the neoplasm originated from the right ovary. Ovariohysterectomy was performed, and the excised tissue was submitted to the Animal Pathology Laboratory of the same institution for histopathological evaluation.

Macroscopically, the mass measured 27.5 × 22.5 × 5.5 cm, with a soft, irregular, reddish surface and a papillary architecture, interspersed with firmer, whitish regions resembling fibrous tissue (Figure 1C). Upon sectioning, a marked amount of blood-tinged fluid was released, and the mass exhibited multifocal cystic areas filled with pale, translucent content. The specimen was fixed in 10% neutral buffered formalin, routinely processed, and stained with hematoxylin and eosin (H&E) for histological examination.

Microscopically, the lesion consisted of a papillary epithelial proliferation supported by fibrovascular stroma, with occasional areas containing smooth muscle bundles (Figure 1D). The papillae were lined by a single to double layer of well-differentiated ciliated columnar epithelial cells, exhibiting eosinophilic cytoplasm, sometimes vacuolated and containing intracytoplasmic granules (Figure 1E). Nuclei were round to oval with inconspicuous nucleoli. Mild anisocytosis, anisokaryosis and nuclear pleomorphism were observed, while mitotic figures were absent. Periodic acid–Schiff (PAS) staining revealed multifocal mild granular eosinophilic cytoplasmic staining. Based on the histomorphological and histochemical features, the lesion was diagnosed as a uterine tube adenoma. Immunohistochemistry was performed with the use of antibodies against Ki-67 (MIB-1, Dako®, ready-to-use), estrogen receptor (ER) (clone EP1, Dako®, ready-to-use), and progesterone receptor (PR) (clone hPRa2, Invitrogen®, 1:50). Immunolabeling for ER and PR was strongly positive in 80% and 90% of neoplastic cells, respectively. For positive control of ER and PR, canine mammary tissue was used, and the negative control was performed by omitting the primary antibody. Ki-67 expression was low, with <10% of positively labeled cells, assessed in a count of 500 cells (Figure 1F-G).

## Discussion

Tumors of the uterine tube are extremely rare in domestic mammals, with few reports available in the veterinary literature, many of them dated (Minoccheri & Meluzzi, 1979; Sailasuta et al., 1989). In women, benign uterine tube tumors are also uncommon, with adenomatoid tumors being the most frequently reported histological type (Shahi et al., 2017). In veterinary medicine, only one case of adenomatoid tumor has been documented in bitches (Gelberg & McEntee, 1986), which highlights the clinical and pathological relevance of the present report.

The main clinical sign observed in this patient was abdominal distension, consistent with previous reports (Marino et al., 2007; Pierezan et al., 2016; Terai et al., 2025). This finding highlights the expansive potential of the tumor, which may reach large dimensions within the abdominal cavity. In addition, the gross observation of papillary projections on the tumor surface has also been described in other reports (Marino et al., 2007; Pierezan et al., 2016; Terai et al., 2025).

Histologically, the presence of papillary projections lined by ciliated columnar epithelium was a key diagnostic feature supporting the identification of uterine tube adenoma (Terai et al., 2025), since within the female reproductive tract, the uterine tube is the only structure composed of this specific epithelial type (Kierszenbaum, 2008; Ross & Pawlina, 2016). The abundance of fibrous stroma with interspersed smooth muscle bundles was also consistent with previously described cases (Marino et al., 2007; Pierezan et al., 2016; Sailasuta et al., 1989). The identification of intracytoplasmic granules in epithelial cells, highlighted by PAS staining, is typically used to demonstrate the presence of glycogen and mucin in different cell types and tissues (Ross & Pawlina, 2016). The absence of mitotic figures is in agreement with previous reports describing low mitotic activity in these tumors (Marino et al., 2007; Pierezan et al., 2016; Terai et al., 2025). This finding was reinforced by the low proliferative index observed with Ki-67 immunohistochemistry, supporting the benign nature of the lesion.

Although even rarer than benign uterine tube tumors in mammals, adenocarcinomas of the uterine tube have been described only once in bitches (Agnew & MacLachlan, 2016). These malignant tumors share a similar histological architecture with adenomas but are distinguished by the presence of metastases (Kennedy et al., 1998). In human pathology, malignant tumors of the uterine tube, ovary, and peritoneum are classified by the World Health Organization based on histological patterns and biological behavior (Kurman et al., 2014). However, determining the exact site of origin is not always possible. It has been suggested that up to 80% of tumors previously classified as high-grade serous carcinomas of the ovary or peritoneum in humans may actually originate from the fimbrial end of the uterine tube, implying that the true incidence of tubal carcinomas may be underestimated (Berek et al., 2021). A similar phenomenon of underreporting may also occur in bitches, considering the limited number of published cases.

Differentiation between tubal and ovarian neoplasms can be challenging, particularly in large masses. In this case, the main differential diagnoses include epithelial ovarian neoplasms, especially adenoma and papillary adenocarcinoma, based on the observed microscopic features characterized by arborizing papillary projections (Agnew & MacLachlan, 2016; Kennedy et al., 1998). However, ovarian carcinomas are often aggressive, leading to carcinomatosis and metastasis (Agnew & MacLachlan, 2016). Additionally, epithelial ovarian neoplasms tend to exhibit more marked cellular variation and may or may not present ciliated epithelium, unlike the present case, which demonstrated a well-differentiated ciliated columnar epithelial lining, with mild anisocytosis, anisokaryosis, and pleomorphism, features consistent with tubal differentiation (Kennedy et al., 1998). In human medicine, primary uterine tube tumors show significant clinical and histomorphological overlap with epithelial ovarian neoplasms and primary peritoneal serous carcinomas, which often makes the precise determination of the tumor site of origin difficult (Berek et al., 2021).

Cytopathological features of uterine tube adenomas in veterinary medicine have not been previously described. In the present case, cytology revealed a cohesive epithelial pattern, composed of columnar cells and the presence of structures consistent with apical cilia, a finding considered highly suggestive of tubal differentiation in histopathological evaluation (Kennedy et al., 1998).

Correlation between cytological and histopathological findings in this case proved useful as a pre-surgical diagnostic support, particularly due to the preservation of epithelial organization and the cellular pattern consistent with the upper reproductive tract. In human medicine, cytology is widely used in neoplasms of the reproductive tract, including tubal carcinomas, and may assist in diagnosis and staging through the detection of neoplastic cells in aspirated samples or effusions (Berek et al., 2021). Although more established in malignant lesions, these findings suggest that cytology may also contribute to the evaluation of such lesions in bitches.

The relationship between sex hormones and the development of oviduct tumors in birds, particularly in hens, has been the subject of increasing interest, as these tumors are more frequently observed in this species (Anjum et al., 1989). One study demonstrated that epithelial tumors arising from a specific region of the avian oviduct express estrogen and progesterone receptors, and their presence was associated with a higher histological grade, suggesting that hormonal regulation may influence tumor pathogenesis (Anjum et al., 1989). In the present case, strong immunopositivity for estrogen and progesterone receptors was also observed, a finding consistent with the hormonal expression described in neoplasms of the female reproductive tract, particularly in canine and human mammary tumors, in which these markers are associated with hormonal sensitivity and tumor biological behavior (Gown, 2008; Torres et al., 2021). Therefore, their interpretation should be performed in conjunction with morphological and histopathological findings, since these markers are not specific for determining histogenetic origin. The microscopic morphological features, particularly the presence of ciliated columnar epithelium, support the neoplastic origin (Kennedy et al., 1998). Nevertheless, data regarding hormonal influence and the molecular mechanisms involved in uterine tube neoplasms in bitches remain scarce.

## Conclusion

In conclusion, although these tumors may reach large dimensions within the abdominal cavity, they are generally amenable to surgical removal, as they typically do not adhere to adjacent organs and are associated with a favorable prognosis. Cytopathological examination may be useful when ciliated epithelial cells are identified, and histopathological features are highly suggestive for establishing a definitive diagnosis.
